# The Estimate for Approximation Error of Neural Network with Two Weights

**DOI:** 10.1155/2013/935312

**Published:** 2013-12-28

**Authors:** Fanzi Zeng, Yuting Tang

**Affiliations:** Key Laboratory for Embedded and Network Computing of Hunan Province, Hunan University, Changsha 410082, China

## Abstract

The neural network with two weights is constructed and its approximation ability to any continuous functions is proved. For this neural network, the activation function is not confined to the odd functions. We prove that it can limitlessly approach any continuous function from limited close subset of *R* ^*m*^ to *R* ^*n*^ and any continuous function, which has limit at infinite place, from limitless close subset of *R* ^*m*^ to *R* ^*n*^. This extends the nonlinear approximation ability of traditional BP neural network and RBF neural network.

## 1. Introduction

So far, many neural networks models have been presented and applied in pattern recognition, automatic control, signal processing, and aided decision. Among these models, BP (feedforward) neural networks and RBF (radial basis function) neural networks are widely used because of their nonlinear ability of approximation to any continuous function. Up to now, these two classes of neural networks have successfully been applied to approach any nonlinear continuous function defined on bounded close subsets [1–7]. However, the approximation result to any continuous function defined on unbounded set has been very rare [8]. This inspires us to look for a new network which is of more wide approximation ability and can approach any continuous function both on a bounded set and on an unbounded set.

Wang et al. [9] presented a new neural network called neural networks with two weights on the base of combining the advantage of BP neural network with that of RBF neural network. This model can not only simulate BP neural network and RBF neural network, but also simulate neural networks with higher order. This neural network not only contains direction weight value with respect to BP network but also contains core weight value with respect to RBF network. The function of its neurons is of the following form:

(1) $$\begin{array}{c} y=\phi \left[\sum_{j=1}^{m}{\left(\frac{\omega_{j}\left(x_{j}-z_{j}\right)}{\left|\omega_{j}\left(x_{j}-z_{j}\right)\right|}\right)}^{s}{\left|\omega_{j}\left(x_{j}-z_{j}\right)\right|}^{p}+\theta \right], \end{array}$$

where *y* is the output of neurons, *ϕ* is the activation function, *θ* is the threshold value, *ω*_*j*_ is the direction weight value, *z*_*j*_ is the core weight value, *x*_*j*_ is the input, and *p* and *s* are two parameters.

In (1), when *z*_*j*_ = 0, *p* = 0, and *s* = 1, then (1) reduces to mathematical model BP network neurons; when *ω*_*j*_ takes a fixed value, *p* = 2 and *s* = 0, then (1) becomes mathematical model RBF network neurons.

In [10], the approximation operators with logarithmic sigmoidal function of a class of neural networks with two weights and a class of quasi-interpolation operators were investigated. Using these operators as approximation tools, the upper bounds of estimate errors were estimated.

In [2], the authors considered single hidden layer neural networks:

(2) $$\begin{array}{c} N_{n}\left(x\right)=c_{0}+\sum_{i=1}^{n}c_{i}\phi \left(\omega_{i}x+\theta_{i}\right),\;N_{n}:R\to R, \end{array}$$

where *c*_*i*_ represents output weights, *ϕ* is the activaion function, *θ*_*i*_ is threshold value, and *ω*_*j*_ is direction weight value.

The main result in [2] is as follows.

**Theorem 1**

Suppose *ϕ* is a bounded, strictly monotonously increasing, and odd function defined on *R*, *f* ∈ *C*[*a*, *b*], and *n* ∈ *N*. Then there exists a feedforward neural network (BP) with one hidden layer *N*_*n*_ → *R* defined by

(3) $$\begin{array}{c} N_{n}\left(x\right)=c_{0}+\sum_{i=1}^{n}c_{i}\phi \left(\omega_{i}x+\theta_{i}\right),\;N_{n}:R\to R, \end{array}$$

such that

(4) $$\begin{array}{c} \underset{x\in \left[a,b\right]}{\sup}\left|N_{n}\left(x\right)-f\left(x\right)\right|\leq \frac{5}{2}\omega \left(f,\frac{b-a}{n}\right), \end{array}$$

where for *i* = 1,2,…, *n*

(5) $$\begin{array}{c} x_{i}=a+i\frac{b-a}{n},\;\;x_{0}=a,\;\;m=\underset{x\in R}{\sup}\;\phi \left(x\right),\\ c_{i}=\frac{1}{2m}\left(f\left(x_{i}\right)-f\left(x_{i-1}\right)\right),\;\;d_{n}=\phi^{-1}\left(m-\frac{m}{2n}\right),\\ c_{0}=f\left(a\right)-\sum_{i=1}^{n}c_{i}\phi \left(\omega_{i}x+\theta_{i}\right),\\ \theta_{i}=-\frac{d_{n}n}{b-a}\left[2a+\left(2i-1\right)\frac{b-a}{n}\right],\;\omega_{i}=\frac{2d_{n}n}{b-a}. \end{array}$$

In this paper, one is concerned with the neural networks with two weights and a single hidden layer:

(6) $$\begin{array}{c} N_{n}\left(x\right)=\sum_{i=1}^{n}c_{i}\phi \left(l_{i}^{s}\frac{{\left(x-z\right)}^{s}}{{\left|\left(x-z\right)\right|}^{s}}{\left|x-z\right|}^{p}-\theta_{i}\right). \end{array}$$

One objective is to construct a new BP neural network which is different from the BP neural network in [2] and prove this network has the approximation ability to any nonlinear continuous function. Another objective is to prove that, by adjusting the values of two parameters *s* and *p*, the neural network with two weights can not only approximate any continuous functions defined on a bounded close subset, namely, it has the same approximation ability as BP neural network in [2], but also approximate any continuous function defined on an unbounded set. Namely, the neural network with two weights has a better approximation ability than BP neural network and RBF neural network.

## 2. Theoretical Result

We use the following notations: the symbols *N*,  *N*_0_, and *R* represent the set of positive integers, nonnegative integers, and real number, respectively. Using *C*[*a*, *b*] to denote the space of continuous real-valued functions defined on [*a*, *b*], it is equalled by the supremum norm ||·||. Let *f* be a real-valued function defined on [*a*, *b*]. *η*(*f*, *δ*), the modulus of continuity of *f*, is defined as *η*(*f*, *δ*) = sup⁡_0<*h*≤*δ*_⁡||*f*(·+*h*) − *f*(·)||. Then we have lim⁡_*δ*→0_⁡*η*(*f*, *δ*) = 0, and for any real number *λ* ≥ 0,  *η*(*f*, *λδ*) ≤ (1 + *λ*)*η*(*f*, *δ*).

The modulus of continuity is usually considered as the measure of the smoothness of function and the approximation error in approximation theory. The function *f* is called Lipschitz *α* (0 < *α* ≤ 1) continuous and is written as *f* ∈ Lip_*C*(*f*)_ *α*, if there exists a constant *C*(*f*) such that *η*(*f*, *δ*) ≤ *C*(*f*)*δ* ^*α*^. *C*(*h*) denotes the positive constant dependents only on *h* and its value may be different at different occurrence.

Our main result is as follows.

**Theorem 2**

Suppose *ϕ* is a bounded, strictly monotonously increasing, and odd function defined on *R*, *f* ∈ *C*[*a*, *b*], and *n* ∈ *N*. Then there exists a feedforward neural network (BP) with one hidden layer *N*_*n*_ → *R* defined by

(7) $$\begin{array}{c} N_{n}\left(x\right)=\sum_{i=1}^{n}c_{i}\phi \left(\omega_{i}x+\theta_{i}\right), \end{array}$$

such that

(8) $$\begin{array}{c} \underset{x\in \left[a,b\right]}{\sup}\left|N_{n}\left(x\right)-f\left(x\right)\right|\leq \left(1+\frac{n+1}{4n}\right)\omega \left(f,\frac{b-a}{n}\right), \end{array}$$

where, for *i* = 1,2,…, *n*,

(9) $$\begin{array}{c} x_{i}=a+i\frac{b-a}{n},\;\;d_{n}=\phi^{-1}\left(\frac{m}{2n}-m\right),\;\;x_{0}=a,\\ m=\underset{x\in R}{\sup}\left|\phi \left(x\right)\right|,\;\;c_{i}=\frac{1}{2m}\left(f\left(x_{i}\right)-f\left(x_{i-1}\right)\right),\\ \omega_{i}=\frac{\left(d_{n}-d_{n}^{*}\right)n}{b-a},\\ \theta_{i}=d_{n}-\frac{\left(d_{n}-d_{n}^{*}\right)n}{b-a}\left[a+\left(i-1\right)\frac{b-a}{n}\right],\\ d_{n}^{*}=\phi^{-1}\left(m-\frac{m}{2n}\right). \end{array}$$

**Proof**

Divide [*a*, *b*] into *n* equal intervals; each has length of (*b* − *a*)/*n* and let *a* = *x*_0_ < *x*_1_ < ⋯<*x*_*n*_ = *b*. For *i* = 1,2,…, *n*, we set *c*_*i*_ = (1/2*m*)(*f*(*x*_*i*_) − *f*(*x*_*i*−1_)), *d*_*n*_ = *ϕ* ^−1^((*m*/2*n*) − *m*), *m* = sup⁡_*x*∈*R*_⁡|*ϕ*(*x*)|, *θ*_*i*_ = *d*_*n*_ − ((*d*_*n*_ − *d*_n_*)*n*/(*b* − *a*))[*a* + (*i* − 1)((*b* − *a*)/*n*)], *d*_n_* = *ϕ* ^−1^(*m* − (*m*/2*n*)),  *ω*_*i*_ = (*d*_*n*_ − *d*_n_*)*n*/(*b* − *a*).

For any *x* ∈ [*a*, *b*], given *n*, we assume that *x* ∈ [*x*_*i*−1_, *x*_*i*_] and note that

(10) $$\begin{array}{c} N_{n}\left(x\right)=\sum_{i=1}^{n}\frac{1}{2m}\left(f\left(x_{i}\right)-f\left(x_{i-1}\right)\right)\phi \left(\omega_{i}x+\theta_{i}\right). \end{array}$$

Fix x* ∈ [*x*_*i*−1_, *x*_*i*_] and choose *f*, so that *N*_*n*_(*x*_i_*) = *f*(*x*_i_*). Let *G*_*i*_(*x*_i_*) = *ϕ*(*ω*_*i*_ *x* + *θ*_*i*_); it follows that

(11) Nn(x)−f(xi∗) =Nn(x)−∑i=1n12m(f(xi)−f(xi−1))Gi(xi∗) =∑i=0n12m(f(xi)−f(xi−1))[Gi(x)−Gi(xi∗)].

There are two possible cases to consider: (i) *x* > *x*_i_*; (ii) *x* ≤ *x*_i_*.

*Case (i)*. When *x* > *x*_i_*, we have, for *i* = 1,2,…, *n*,

(12) 0<Gi(x)−Gi(xi∗)<Gi(xi)−Gi(xi∗)<ϕ(ωi(a+ib−an)+θi)+m=ϕ(dn)+m=m2n.

*Case (ii)*. When *x* ≤ *x*_i_*, we have, for *i* = 1,2,…, *n*,

(13) 0>Gi(x)−Gi(xi∗)>Gi(xi−1)−m=ϕ(ωi(a+(i−1)b−an)+θi)−m=ϕ(dn∗)−m=−m2n.

From (12) and (13), it follows that

(14) $$\begin{array}{c} \left|G_{i}\left(x\right)-G_{i}\left(x_{i}^{*}\right)\right|\leq \frac{m}{2n},\;i=1,2,\ldots ,n. \end{array}$$

Thus from (14), we have

(15) Nn(x)−f(xi∗) ≤∑i=0n12m|f(xi)−f(xi−1)||Gi(x)−Gi(xi∗)| ≤n+12mη(f,b−an)×m2n =n+14nη(f,b−an).

Therefore, we have

(16) |Nn(x)−f(x)|≤|Nn(x)−f(xi∗)|+|f(x)−f(xi∗)|=(1+n+14n)η(f,b−an).

This ends the proof of Theorem 2.

**Remark 3**

The activation functions are assumed to be odd functions in [2], while in this paper, this assumption is removed. On the other hand, in the construction of neural networks, threshold values and direction weight values are different from those in [2].

**Theorem 4**

Suppose *ϕ* is a bounded, strictly monotonously increasing, and odd function defined on *R*, *f* ∈ *C*[*a*, *b*], and *n* ∈ *N*, *z* ∉ [*a*, *b*]. Then there exists a neural network with two weights and one hidden layer *N*_*n*_ : [*a*, *b*] → *R* defined by

(17) $$\begin{array}{c} N_{n}\left(x\right)=\sum_{i=1}^{n}c_{i}\phi \left(l_{i}^{s}\frac{{\left(x-z\right)}^{s}}{{\left|\left(x-z\right)\right|}^{s}}{\left|x-z\right|}^{p}-\theta_{i}\right) \end{array}$$

with *p* < *s*, *p* < 0 such that

(18) $$\begin{array}{c} \underset{x\in [a,b]}{\sup}\left|N_{n}\left(x\right)-f\left(x\right)\right|\leq \left(1+\frac{n+1}{4n}\right)\eta \left(f,\frac{b-a}{n}\right), \end{array}$$

where *c*_*i*_ is defined in Theorem 2 and *l*_*i*_^*s*^ is equal to *ω*_*i*_ in Theorem 2.

**Proof**

We make fractional transformation *H* : *x* → *u*, where *u* = (*x*−*z*)^*s*^/|*x*−*z*|^*p*−*s*^, *x* ∈ [*a*, *b*]. Since *z* ∉ [*a*, *b*], thus *H* is a continuous transformation. Hence *f*([*a*, *b*]) = *U* is also a bounded close set; moreover, 0 ∉ *U*.

There exists an inverse transformation for fractional transformation *H* ^−^ : *u* → *x*, where *x* = *z* + *u* ^1/*s*^|*u*|^(*s* − *p*)/*sp*^, *u* ∈ *U*. So *g*(*u*) = *f*(*x*) = *f*(*z* + *u* ^1/*s*^|*u*|^(*s* − *p*)/*sp*^) is a continuous real-valued function defined on bounded close set *U*. For *g*(*u*), from Theorem 2, there exists a neural network with two weights and one hidden layer *N*_*n*_ : [*a*, *b*] → *R* defined by

(19) $$\begin{array}{c} N_{n}\left(u\right)=\sum_{i=1}^{n}c_{i}\phi \left(l_{i}^{s}u-\theta_{i}\right) \end{array}$$

such that

(20) g^(u)=∑i=1nciϕ(lisu−θi)=∑i=1nciϕ(lis(x−z)s|(x−z)|s|x−z|p−θi)=f^(x)

which satisfies

(21) $$\begin{array}{c} \sup\left|g\left(u\right)-\hat{g}\left(u\right)\right|\leq \left(1+\frac{n+1}{4n}\right)\eta \left(f,\frac{b-a}{n}\right). \end{array}$$

This ends the proof of Theorem 4.

**Remark 5**

When *p* < *s*, *p* < 0, the network with two weights is not BP and RBF neural network; thus, Theorem 4 validates that the neural network with two weights has the same approximation ability as BP and RBF neural network on any bounded close set. When *n* → *∞*, from Theorem 4, this neural network can approximate any continuous function defined on a bounded close set.

**Theorem 6**

Suppose *ϕ* is a bounded, strictly monotonously increasing, and odd function defined on *R*; *D* is an unbounded subset of *R* (all accumulation points are in *D* beside *∞*) and*f* ∈ *C*(*D*), *z* ∉ *D*, lim⁡_*x*→*∞*_⁡*f*(*x*) = *b*(*constant*) and then there exists a neural network with two weights and one hidden layer:

(22) $$\begin{array}{c} N_{n}\left(x\right)=\sum_{i=1}^{n}c_{i}\phi \left(l_{i}^{s}\frac{{\left(x-z\right)}^{s}}{{\left|\left(x-z\right)\right|}^{s}}{\left|x-z\right|}^{p}-\theta_{i}\right) \end{array}$$

with *p* < *s*, *p* < 0 such that

(23) $$\begin{array}{c} \hat{f}\left(x\right)=\sum_{i=1}^{n}c_{i}\phi \left(l_{i}^{s}\frac{{\left(x-z\right)}^{s}}{{\left|\left(x-z\right)\right|}^{s}}{\left|x-z\right|}^{p}-\theta_{i}\right) \end{array}$$

which satisfies

(24) $$\begin{array}{c} \underset{x\in [a,b]}{\sup}\left|\hat{f}\left(x\right)-f\left(x\right)\right|<\left(1+\frac{n+1}{4n}\right)\eta \left(f,\frac{b-a}{n}\right). \end{array}$$

**Proof**

We make fractional transformation *H* : *x* → *u*, where *u* = (*x*−*z*)^*s*^/|*x*−*z*|^*p*−*s*^, *x* ∈ *D*. Since *z* ∉ *D*, thus *H* is a contimuous transformation on *D* and as *x* → *∞*, *u* → 0. Let *H*(*∞*) = 0; *H*(*x*) is a continuous function on *D* ∪ {*∞*}. We prove that *H*(*D* ∪ {*∞*}) = *H*(*D*)∪{0} is a close set. If *v* is an arbitrary accumulation point of *H*(*D*)∪{0}, then *v* = 0 or *v* is an accumulation point of *H*(*D*). When *v* = 0, *z* ∈ *H*(*D*)∪{0}. When *v* is an accumulation point of *H*(*D*), then there exists a sequence *u* ^*k*^ ∈ *H*(*D*) and another sequence *x* ^*k*^ ∈ *D*, such that *x* ^*k*^ → *u* ^*k*^,  *u* ^*k*^ → *v* ≠ 0  (*k* → *∞*). From fractional transformation, there exists $\overset{-}{u},z=\overset{-}{u},x^{k}\to \overset{-}{x}$ such that $u^{k}\to \overset{-}{u}$. From *u* ^*k*^ → *v* ≠ 0  (*k* → *∞*) and inverse transformation *H* ^−1^ : *x* = *z* + *u* ^1/*s*^|*u*|^(*s* − *p*)/*sp*^, *u* ∈ *U*, it follows that *x* → *∞* does not hold. Hence $\overset{-}{x}$ is a finite point of *D*. Therefore $\overset{-}{x}\in D$, $v=\overset{-}{u}\in H(D)$. So, *H*(*D* ∪ {*∞*}) = *H*(*D*)∪{0} is a close set. Let

(25) $$\begin{array}{c} g\left(u\right)=f\left(x\right)=f\left(z+u^{1/s}{\left|u\right|}^{\left(s-p\right)/sp}\right). \end{array}$$

Then *g*(*u*) is a continuous function on *H*(*D*). Let *g*(0) = *b* = lim⁡_*x*→*∞*_⁡*f*(*x*); it is easy to prove that *g*(*u*) is a continuous function on *H*(*D*)∪{0}. For a continuous function *g*(*u*) defined on *H*(*D*)∪{0}, by means of Theorem 4, there exists a neural network with two weights ∑_*i*=1_^*n*^ *c*_*i*_ *ϕ*(*l*_*i*_^*s*^((*x*−*z*)^*s*^/|(*x*−*z*)|^*s*^)|*x*−*z*|^*p*^ − *θ*_*i*_) such that

(26) g^(u)=∑i=1nciϕ(ωi(x−z)s|(x−z)|s|x−z|p−θi)=f^(x)

which satisfies

(27) $$\begin{array}{c} \sup\left|g\left(u\right)-\hat{g}\left(u\right)\right|\leq \left(1+\frac{n+1}{4n}\right)\eta \left(f,\frac{b-a}{n}\right). \end{array}$$

This ends the proof of Theorem 6.

**Remark 7**

When *p* < *s*,  *p* < 0, the network with two weights is not BP and RBF neural network; thus, Theorem 6 validates that the neural network with two weights has the approximation ability that BP and RBF neural network do not have on any unbounded set. Namely, the network with two weights has a better approximation ability than BP and RBF neural network on any unbounded set. When *n* → *∞*, from Theorem 6, this neural network can approximate any continuous function defined on unbounded set.

## 3. Conclusions

In this paper, we construct a new BP neural network and prove that the network has the approximation ability to any nonlinear continuous function. In our result, the threshold values and direction weight values in [2] are different from those in our paper. Second, we prove that neural network with two weights has the same approximation ability as BP neural network and RBF neural network to any continuous function defined on any bounded close set; furthermore, we prove that neural network with two weights has a better approximation ability than BP neural network and RBF neural network to any continuous function defined on any unbounded set.
